# The Application of Globular Water-Atomized Iron Powders for Additive Manufacturing by a LENS Technique

**DOI:** 10.3390/ma11050843

**Published:** 2018-05-18

**Authors:** Tomasz Durejko, Justyna Aniszewska, Michał Ziętala, Anna Antolak-Dudka, Tomasz Czujko, Robert A. Varin, Vlad Paserin

**Affiliations:** 1 Faculty of New Technologies and Chemistry, Military University of Technology, 2 Urbanowicza Str., 00-908 Warsaw, Poland; justyna.aniszewska@wat.edu.pl (J.A.); michal.zietala@wat.edu.pl (M.Z.); anna.dudka@wat.edu.pl (A.A.-D.); tomasz.czujko@wat.edu.pl (T.C.); 2 Department of Mechanical and Mechatronics Engineering and Waterloo Institute for Nanotechnology, University of Waterloo, 200 University Ave. W., Waterloo, ON N2L 3G1 Canada; robert.varin@uwaterloo.ca; 3 Rio Tinto Metal Powders, 1655 Route Marie-Victorin, Sorel-Tracy, QC J3R 4R4 Canada; vlad.paserin@uwaterloo.ca

**Keywords:** additive manufacturing, LENS technique, globular powder, water atomization

## Abstract

The water-atomized ATOMET 28, 1001, 4701, and 4801 powders, manufactured by Rio Tinto Metal Powders, were used for additive manufacturing by a laser engineered net shaping (LENS) technique. Their overall morphology was globular and rounded with a size distribution from about 20 to 200 µm. Only the ATOMET 28 powder was characterized by a strong inhomogeneity of particle size and irregular polyhedral shape of powder particles with sharp edges. The powders were pre-sieved to a size distribution from 40 to 150 µm before LENS processing. One particular sample—LENS-fabricated from the ATOMET 28 powder—was characterized by the largest cross-sectional (2D) porosity of 4.2% and bulk porosity of 3.9%, the latter determined by microtomography measurements. In contrast, the cross-sectional porosities of bulk, solid, nearly cubic LENS-fabricated samples from the other ATOMET powders exhibited very low porosities within the range 0.03–0.1%. Unexpectedly, the solid sample—LENS-fabricated from the reference, a purely spherical Fe 99.8 powder—exhibited a porosity of 1.1%, the second largest after that of the pre-sieved, nonspherical ATOMET 28 powder. Vibrations incorporated mechanically into the LENS powder feeding system substantially improved the flow rate vs. feeding rate dependence, making it completely linear with an excellent coefficient of fit, R^2^ = 0.99. In comparison, the reference powder Fe 99.8 always exhibited a linear dependence of the powder flow rate vs. feeding rate, regardless of vibrations.

## 1. Introduction

The emerging technology of metal additive manufacturing (metal 3D printing) creates demand for high-quality, economical metal powders used as feedstock material in 3D metal printers. Historically, powders with spherical particle morphology produced by gas and plasma-assisted atomization have been considered as most suitable for this application. More recently, water-atomized powders are being evaluated in 3D metal printers for additive manufacturing, raising a question about the strict requirement for spherical particle morphology [1] and paving the way to economical (water-atomized Fe powders are approximately 10 times cheaper than the gas-atomized ones), mass-produced metal powder feedstock materials [2]. More specifically, water-atomized low-alloy steel and iron powders represent a unique category of metal powders that are economically mass-produced to meet the needs of the large ferrous powder metallurgy market serving primarily the automotive industry [3].

The synthesis of iron powders employed in this study was carried out by water atomization of molten iron using the proprietary Rio Tinto process [4]. It was recently reported that water-atomized iron powders could be used to additively manufacture components with a final density of up to 99.8% by laser powder bed fusion (L-PBF)/selective laser melting [5].

The specific objective of this work was to characterize, morphologically and chemically, four batches of water-atomized powders in comparison to the commercially available spherical powder. Furthermore, the fully metallurgically characterized powders, pre-sieved to a specific size range, which are nonspherical (globular), were used for fabrication of solid cubic samples by the LENS (laser engineered net shaping) fabrication technique in order to find out whether or not the nonspherical, but still globular, powders would be suitable for fabrication by the LENS technique of microstructurally sound, solid samples with a minimized porosity level. For comparison, a fully spherical iron powder purchased from TLS Technik was also employed for LENS-fabricating similar solid samples.

## 2. Materials and Methods

Four different types of commercially made, water-atomized, nonspherical powders were supplied by Rio Tinto Metal Powders, Montreal, Canada (designated ATOMET 28, 1001, 4701, and 4801). In order to verify the necessity of using spherical powders in the LENS process, the producer-recommended spherical reference gas-atomized powder Fe 99.8 (TSL Technik, Bitterfeld, Germany, 99.8 purity, 40–150 µm) was used. The chemical composition of investigated powders, based on the data provided by the manufacturer, is presented in Table 1. The storage and handling of all powders with the powder feeders was conducted in a Labmaster Glovebox Workstation (MBraun, Garching, Germany) under continuously supplied pure argon (purity 99.9999%).

OPTOMEC recommends that the powder particle sizes for an effective LENS fabrication technique fall within the optimal range of 40–150 μm [6]. Due to the fact that only a certain size fraction of each of the as-received ATOMET powders was within that recommended range, the as-received powders were pre-sieved to the optimal range of 40–150 μm. A sieving system produced by Fritsch (Idar-Oberstein, Germany) was used, with sieving time of 10–13 min at the amplitude of 1.0–1.3 mm. The particle powder size analysis for pre-sieved powder was carried out using an IPS UA particle size analyzer (KAMIKA, Warsaw, Poland). The volume fraction (volume %) of equivalent particle diameter was measured in the range of 0–200 μm at with the frequency of ultrasound dispenser equal to 40 kHz.

The analysis was carried out for at least 10,000 counts. Furthermore, the pre-sieved ATOMET powders were compared to a purely spherical, reference, pure iron powder from TLS Technik (Bitterfeld, Germany) having a declared purity of 99.8% and particle size distribution within a 40–150 µm range.

The powder morphology observations and chemical analysis were carried out on loose powders attached to sticky carbon electrical conductive tape. The morphology evaluation of the initial powder was carried out using an FEI Quanta 3D scanning electron microscope (SEM) field emission gun scanning electron microscope (FEG-SEM; FEI, Hillsboro, OR, USA).

The porosity was evaluated by quantitative image analysis at a low magnification (10×) using a Nikon Eclipse MA2000 light microscope (Nikon Instruments Europe, Amsterdam, The Netherlands) equipped with a CCD camera. The porosity was determined as the area fraction of pores relative to the area of the whole vertical cross-section of samples. The image of the whole vertical cross-section was prepared by assembling sequential images (50× magnifications) of particular areas of a sample in the x–z plane to show all layers from the substrate to the top of the deposit. Macroscopic and microscopic observations were conducted across entire sections of the samples. Before structural examinations, all samples were subjected to the following metallographic preparation: grinding on SiC papers with granulations of 120, 240, 500, 1200, and 2400, in sequence, polishing with diamond suspensions (3 µm, 1 µm, and 0.25 µm) and, finally, polishing with silica suspensions of 0.1 µm and 0.06 µm.

Moreover, the reconstruction of samples scanned with 20 µm voxel size using a microfocus X-ray computer tomography (µCT) Nikon/METRIS XT H 225 ST (Nikon Instruments Europe, Amsterdam, The Netherlands) were taken to calculate porosity and observe the distribution of pores.

**Figure 1:**
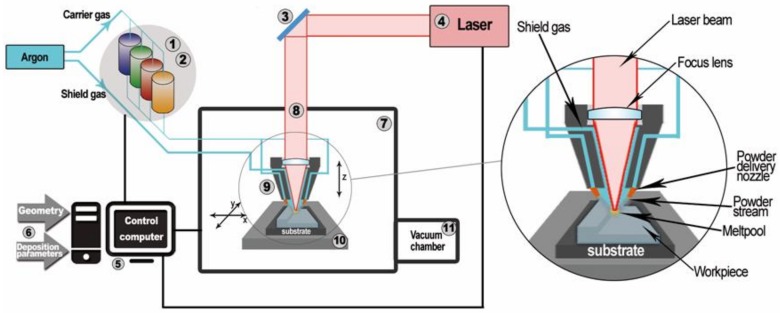
Scheme of the LENS (laser engineered net shaping) system [7]: 1. Powder supply—it is possible to install and use a supply unit with up to four powders; 2. Pneumatic turbine vibrators that provides stable powder doses; 3. Optical system for focusing the laser beam, the optical path, and the imager thermal system for analyzing the temperature distribution of the liquid metal; 4. IPG fiber laser with a wavelength of 1070 nm and power of 500 W; 5. Computers for process control; 6. Input data entered by the operator; 7. Working chamber with a protective atmosphere with controlled oxygen and water vapor content (less than 10 ppm). Work in the chamber was conducted using butyl gloves and sight glass in the form of transparent plastic with a filter to protect the operator from high-power laser radiation; 8. Optical path of the laser; 9. Working head with four nozzles controlling the flow of powder into the laser beam focus zone; 10. Numerically controlled working table (movement in the X–Y plane); 11. Vacuum chamber.

After detailed analysis of the investigated powders, fabrication of the solid samples was performed using the LENS MR-7 system (OPTOMEC, Albuquerque, NM, USA). A schematic diagram and the working principles of the LENS MR-7 system that was used in the present work is shown in Figure 1 [7]. The device melts the supplied material, in the form of metal or ceramic powders, and selectively deposits on the substrate or the previously built layer using a 0.5 kW continuous wave IPG YLR-500 fiber laser (IPG Photonics, Oxford, MA, USA) [8]. This technology allows the simultaneous formation of a microstructure and geometry of the manufactured component through the precise control of the working parameters, such as the powder flow rate, the laser power, the feed of the working table, and the heat transfer rate.

The LENS manufacturing process starts with a solid CAD model that is sliced in the next step using PartPrep software (Version 2.2.11, OPTOMEC, Albuquerque, NM, USA). Based on the prepared slice file, the tool path is generated and allows the operator to build the metal parts during the final step of the deposition process. The LENS control software allows the determination of the deposition parameters, such as powder flow rate, layer thickness, laser power, or acceleration and deceleration of the laser working table during the building process.

The samples selected for further investigation were selected with respect to surface roughness and waviness. The measurements were done on the lateral surface from the bottom to the top, as well as on the top surface in the direction perpendicular to the direction of last layer deposition. Each surface was measured 10 times. The measurement length was always 8 mm, in accordance to the ISO 4288 standard. The average value of Ra and Wa parameters +/− standard deviation was calculated with IOS Topografia software (Version 1.4.1.183, Cracow, Poland). The measurements were carried out using contact a PGM-1C profilometer made by IOS (Cracow, Poland).

In order to assess the uniformity of powder flow through the feeding nozzles, which direct the feeding powder into the working area of the laser beam, the calibration characteristics, such as powder flow rate (g/min) and feeding rate (RPM) with and without vibrations, were established. The vibrations were induced by a pneumatic turbine vibrator (Findeva, Oerlingen, Switzerland) mounted on the bottom side of the powder feeder. The air pressure powered the turbine was 1 bar. The powder flow rate vs. feeding rate analysis was carried out with the Alloy Development System (Version 1.0, Faculty of New Technologies and Chemistry, Military University of Technology, Warsaw, Poland) combined with an analytical balance WPS600/2 (Radwag, Radom, Poland).

## 3. Results and Discussion

### 3.1. Powder Characterization

**Figure 2:**
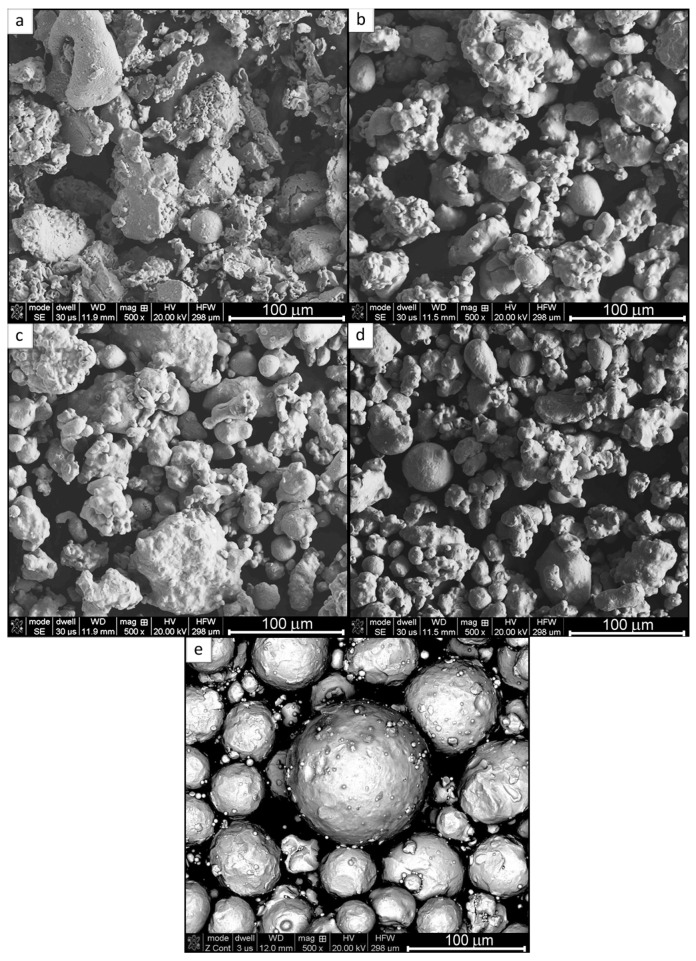
Secondary electron SEM micrographs of the morphology of the pre-sieved ATOMET water-atomized powders: (**a**) 28; (**b**) 1001; (**c**) 4701; (**d**) 4801; and (**e**) the reference globular Fe 99.8.

Figure 2a,b show the SEM micrographs of the morphology of the ATOMET 28 powder. It is characterized by a strong inhomogeneity of particle size and irregular polyhedral shape of powder particles.

The morphology of the ATOMET 1001 powder in Figure 2b is characterized by a relatively homogenous particle size distribution and a rounded/globular particle shape without sharp edges. Moreover, single spherical particles are also observed. The ATOMET 4701 powder is characterized by a shape similar to that of ATOMET 1001. However, the coarse and fine fractions of powder are observed. Figure 2d shows the SEM micrographs of the morphology of the ATOMET 4801 powder. In general, the powder is relatively homogeneous. The morphology is characterized by a rounded, globular shape and highly developed surface. In addition, single spherical particles with the size of ~30 µm are also observed. Figure 2e is an SEM micrograph showing the purely spherical morphology of the reference Fe 99.8 powder.

**Figure 3:**
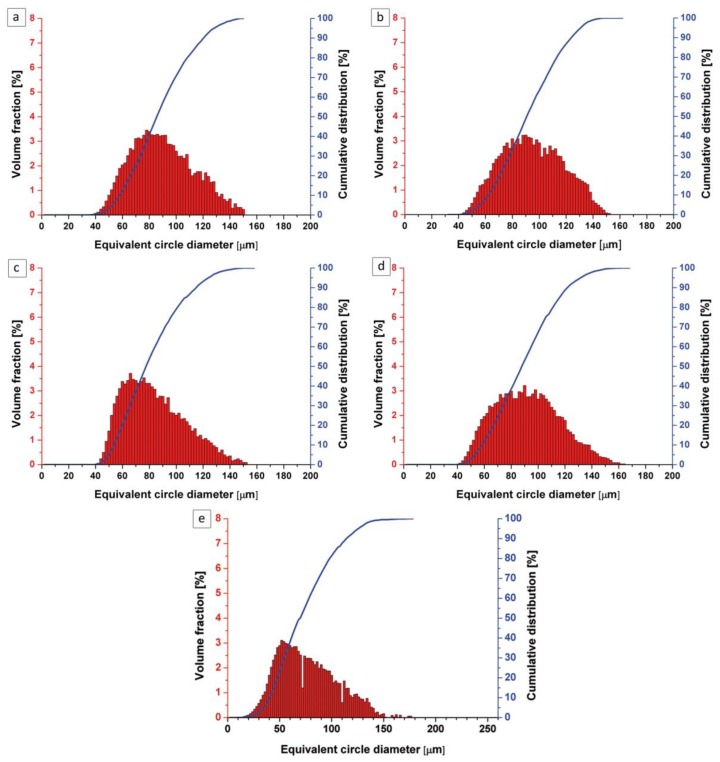
Particle size distribution of the pre-sieved, water-atomized, nonspherical ATOMET powders: (**a**) 28; (**b**) 1001; (**c**) 4701; and (**d**) 4801 and (**e**) the spherical Fe 99.8 reference powder.

Figure 3 shows a particle size distribution for the pre-sieved ATOMET powders and the spherical Fe 99.8 reference powder. It is clearly seen that pre-sieving succeeded in refining the particle size distribution to the desired range of 40–150 µm. The exact volume fractions for the pre-sieved particles within the 40–150 µm range, for all powders, were found to be as follows: ATOMET 28–99.87%, ATOMET 1001–100%, ATOMET 4701–99.9%, ATOMET 4801–99.24%.

### 3.2. The Role of Vibration in the Powder Feed System

**Figure 4:**
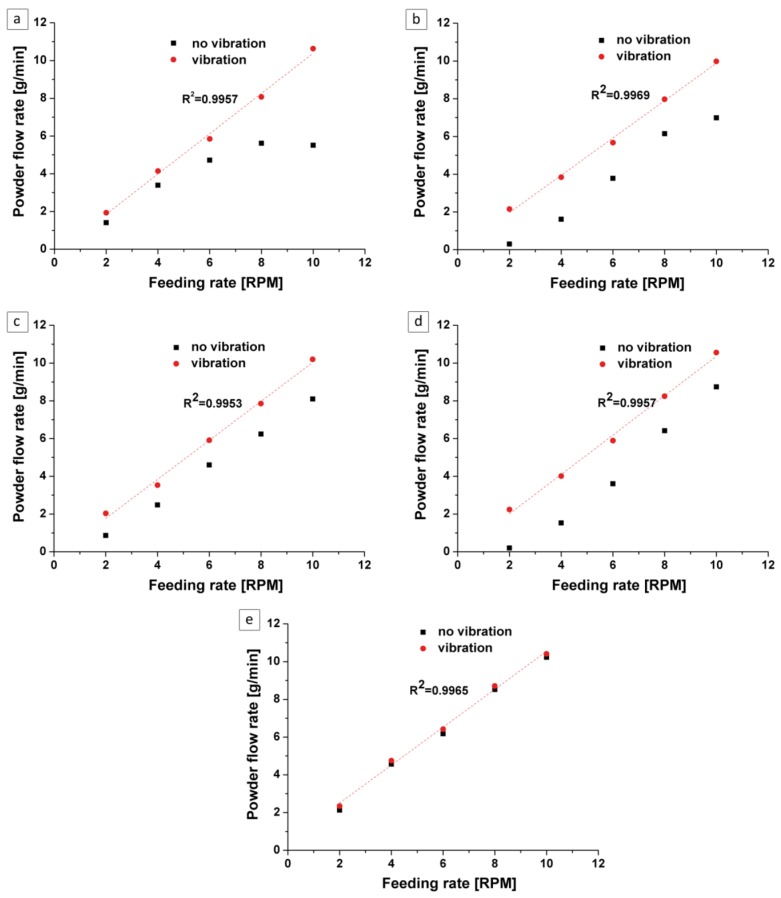
Powder flow rate (g/min) vs. feeding rate (RPM) without and with vibrations for the nonspherical ATOMET powders: (**a**) 28; (**b**) 1001; (**c**) 4701; and (**d**) 4801, as compared to (**e**) the reference, purely spherical powder Fe 99.8 The particle size range for all powders was within 40–150 µm.

In order to assess the uniformity of the powder flow, which influences the proper dimensionality of the bulk samples during LENS fabrication, the calibration characteristics—such as powder flow rate (g/min) vs. feeding rate (RPM)—were analyzed for each ATOMET, as well as the reference Fe 99.8 powder. The analysis was carried out without and with vibrations in the feeder system implemented by an eccentric turbine powered by compressed air at 1 bar. It was found that for the ATOMET 28 powder fed without vibrations, there was a tendency (especially at higher RPM) for agglomerating at the channel leading to a nozzle, with the effect of reducing powder flow rate as shown in Figure 4a (square data points).

Furthermore, for the ATOMET 1001, 4701, and 4801 powders fed without vibrations, the powder flow rate went down to zero at a feeding rate of 2 RPM (Figure 4b–d, solid square data points). All these detrimental effects were completely eliminated after incorporating vibrations into the system after which the dependence of flow rate (g/min) vs. feeding rate (RPM) became linear, exhibiting very high coefficients of fit, R^2^, as shown in Figure 4a–e (circle data points). It is interesting to point out that the reference, spherical Fe 99.8 powder showed a linear dependence of flow rate vs. feeding rate regardless of whether or not vibrations were incorporated into the system. Apparently, the spherical shape of the powder seems to be a principal prerequisite for a smooth feeding process during LENS fabrication.

### 3.3. Characterization of Fabricated Samples

After powder analysis, sets of approximately 10 × 10 × 10 mm nearly cubic samples were fabricated from each powder, using the LENS MR-7 system. The set of parameters for the investigated powders are compiled in Table 2.

**Figure 5:**
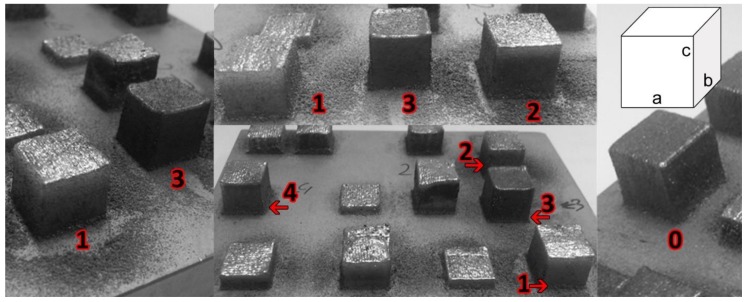
Nearly cubic samples with dimensions a, b, and c were fabricated by the LENS technique from the pre-sieved ATOMET. The numerals 0, 1, 2, 3, and 4 designate the specific material used in a given printing test, namely, the reference Fe 99.8, ATOMET 28, 1001, 4701, and 4801 powder, respectively.

The samples fabricated from ATOMET 28, 4701, and 4801, as well as the reference Fe 99.8 powder, were nearly cubic (Figure 5) with all three dimensions very close to 10.5 mm (Table 3).

The geometrical quality of the surfaces of the fabricated, nearly cubic samples were assessed by measuring the surface roughness and waviness on the lateral surface from the bottom to the top and on the top surface in the direction perpendicular to the direction of the last layer deposition. The results are presented in Table 4.

It is observed in Table 4 that the roughness (Ra) of the top surfaces is, on average, higher than that for the lateral surfaces for all samples fabricated from the pre-sieved ATOMET powders. Interestingly, the lateral and top surface roughnesses (Ra) for the reference sample, fabricated from a purely spherical Fe 99.8% powder, are relatively high. In turn, the values of waviness (Wa) for the lateral and top surface for the reference Fe 99.8 sample are relatively even at the 4–5 µm range, as compared to the pre-sieved LENS-fabricated ATOMET 1001, 4701, and 4801 samples, which exhibit a substantial difference between waviness (Wa) for the lateral and top surfaces. Surprisingly, the waviness of the ATOMET 28 sample is very similar for the lateral and top surface and, on average, slightly smaller than that for the reference Fe 99.8 sample.

**Figure 6:**
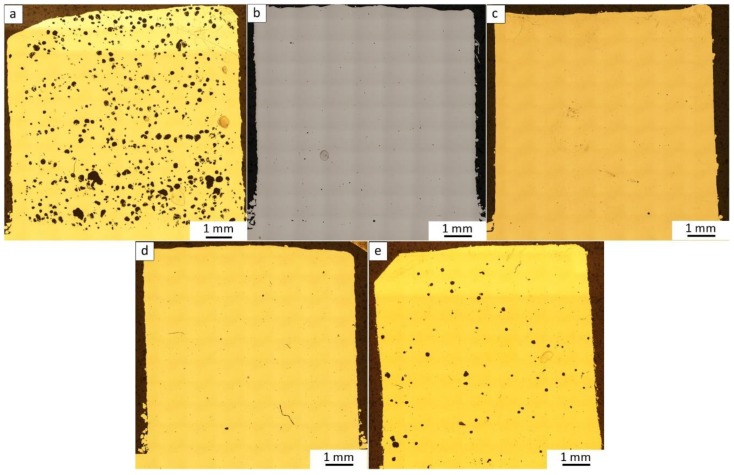
The macrostructure (2D) of the LENS-fabricated bulk samples from (**a**) ATOMET 28; (**b**) ATOMET 1001; (**c**) ATOMET 4701; and (**d**) ATOMET 4801 powders and (**e**) spherical Fe 99.8 reference powder obtained by polishing of metallurgical cross-sections and optical imaging.

The cross-sectional (2D) porosity of the nearly cubic samples of the LENS-fabricated ATOMET powders was investigated (Figure 6). The results of porosity measurements are listed in Table 5. The highest porosity of 4.2% is exhibited by the ATOMET 28 LENS-fabricated sample. Surprisingly, the purely spherical reference Fe 99.8 sample has a relatively high porosity of 1.1%. Apparently, the spherical shape alone is not conducive to obtaining a minimal porosity in a LENS-fabricated sample. At this point, it is necessary to mention that the parameters of the cubic element manufacturing process with spherical powder were optimized in order to obtain the highest geometric compatibility of the produced element. Moreover, the porosity at the measured range is rather typical for the elements fabricated with pure Fe. Montani et al. [9] obtained an element made of Fe, using an SLM (Selective Laser Melting) technique, with a porosity from 7.9 to less than 1%, depending on the fluence.

**Figure 7:**
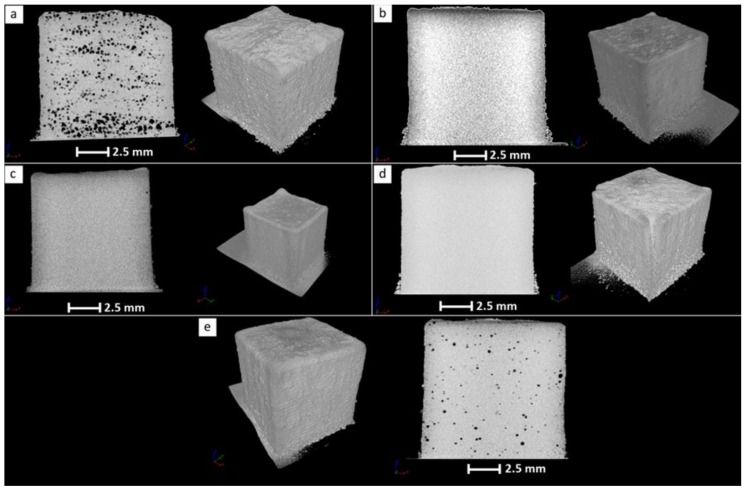
A microfocus X-ray computer tomography (µCT) image of the LENS-fabricated sample from the pre-sieved ATOMET 28 (**a**); ATOMET 1001 (**b**); ATOMET 4701 (**c**); ATOMET 4801 and (**d**); nonspherical powder and (**e**) from the reference Fe 99.8 purely spherical powder.

The presence of bulk porosity was also assessed by the reconstruction of a number of sample cross-sections using microfocus X-ray computer tomography (µCT). The µCT images for the LENS-fabricated bulk samples from the ATOMET 28, 1001, 4701, 4801, and reference Fe 99.8 powders are shown in Figure 7.

The µCT images in Figure 7 confirm qualitatively that, indeed, the LENS-fabricated sample from ATOMET 28 powder has the highest level of bulk porosity, estimated from the images as being at the level of 3.9%. Also, it is confirmed that the porosity of the LENS-fabricated sample from the reference Fe 99.8 powder has the second highest porosity of 0.9%, as estimated from the µCT images. It is also confirmed that none of the LENS-fabricated bulk samples exhibit any microcracks.

**Figure 8:**
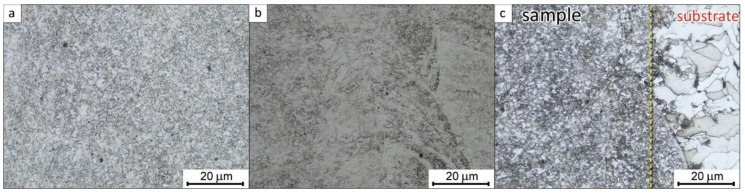
Optical micrographs of an example of LENS-fabricated solid cubic sample from the pre-sieved ATOMET 4701 powder. (**a**) Surface area of deposited sample; (**b**) center/bulk of deposited sample; and (**c**) sample/substrate area. These images represent the microstructure typical of samples made with the as-received and pre-sieved ATOMET powders.

Figure 8 shows optical micrographs of the typical microstructure for solid samples fabricated by the LENS technology. This particular micrograph shows the microstructure of a sample LENS fabricated from the pre-sieved 4701 ATOMET powder. The grain structure at the surface of the solid sample is very fine (Figure 8a). The average grain size is roughly about 10 µm. Some parts of the central portion of the sample (Figure 8b) still contain a characteristic “layered” structure resulting from the sequential layer method of building a solid sample by the LENS machine. This section contains some larger, irregular grains. Finally, the section of the sample near the substrate (Figure 8c) is very fine-grained, with the average grain size similar to that of the surface section.

It must be pointed out that the microstructures of the samples fabricated from the pre-sieved ATOMET powders were essentially identical.

## 4. Conclusions

1. The four, water-atomized, nonspherical ATOMET powders—designated as ATOMET 28, 1001, 4701, and 4801—were characterized by an overall rounded, globular morphology and relatively wide particle size distribution from 20 to 200 µm.
2. As an exception, the ATOMET 28 powder was characterized by a strong inhomogeneity of particle size and irregular polyhedral shape of powder particles with sharp edges.
3. Before the LENS processing of solid samples, all four ATOMET powders were pre-sieved to the size range 40–150 µm.
4. The nearly cubic solid sample from the LENS-fabricated ATOMET 28 powder exhibited the largest cross-sectional (2D) porosity of 4.2% and bulk porosity of 3.9%, the latter obtained from the microtomography measurements. This is most likely due to the strong inhomogeneity of particle size and irregular polyhedral shape of powder particles with sharp edges present in this particular powder. In contrast, the cross-sectional porosities of bulk, solid, nearly cubic samples from the other LENS-fabricated ATOMET powders exhibited very low porosities within the range 0.03–0.1%.
5. Surprisingly, the solid sample from the LENS-fabricated reference, purely spherical Fe 99.8 powder, exhibited the second largest porosity of 1.1%, after that of the pre-sieved, nonspherical ATOMET 28 powder.
6. Incorporation of vibrations into the LENS powder feeding system substantially improved the flow rate vs. feeding rate dependence, making it completely linear with excellent coefficients of fit R^2^ = 0.99.
7. Interestingly, the reference powder Fe 99.8 always exhibited a linear dependence between the flow rate vs. feeding rate regardless of whether or not vibrations were incorporated into the powder feeding system. Apparently, a purely spherical powder eliminates any LENS feeding problems.

## Figures and Tables

**Table 1:** The chemical composition of ATOMET powders.

ATOMET Powder	Element (wt %)							
	Fe	C	O	S	Mn	Mo	Ni	Cr
28	Bal.	0.042	0.110	0.011	na	na	na	na
1001	Bal.	0.004	0.070	0.011	0.169	na	na	na
4701	Bal.	0.014	0.170	0.009	0.447	1.011	0.897	0.471
4801	Bal.	0.005	0.070	0.006	0.137	0.537	3.945	na
Fe 99.8	Bal.	0.050	0.120	n/a	n/a	n/a	n/a	n/a

**Table 2:** Selected technological variants for manufacturing cubic samples made from the ATOMET powders.

		ATOMET 28	ATOMET 1001	ATOMET 4701	ATOMET 4801	Fe 99.8
**Number of coils**		3	3	3	3	3
**Gas (L/min)**	**Laser**	40	40	40	40	40
	**Powder**	2.8	2.8	2.8	2.8	2.8
**Power (W)**		300	300	300	300	300
**Feeding rate (RPM)**		6	6	6	6.2	4.6
**Travel (mm/s)**	**Contour**	10.5	9.5	9.5	9.5	11.5
	**Filling**	15	15	15	15	15
	**Set up movement**	10	10	10	10	10
**Layer thickness (mm)**		0.2	0.2	0.2	0.2	0.2
**Hatch**	**deg**	0°/90°	0°/90°	0°/90°	0°/90°	0°/90°
	**(mm)**	0.3	0.35	0.3	0.3	0.3
**Hatch shrink**		0.05	0.05	0.05	0.05	0.05
**Vibration**		1 bar, 45°	1 bar, 45°	1 bar, 45°	1 bar, 45°	1 bar, 45°
**Remarks**		Acc/dec: 40,000	Acc/dec: 40,000	Acc/dec: 40,000	Acc/dec: 40,000	Acc/dec: 40,000

Acc/dec: acceleration/deceleration.

**Table 3:** Dimensions of LENS-fabricated samples.

ATOMET Powder	a (mm)	b (mm)	c (mm)
28	10.51	10.55	10.52
1001	10.47	10.40	10.42
4701	10.45	10.40	10.47
4801	10.49	10.41	10.50
Fe 99.8	10.53	10.50	10.47

**Table 4:** The summary of roughness (Ra) and waviness (Wa) of lateral and top surface for the samples fabricated with the pre-sieved ATOMET powders and the spherical Fe 99.8 reference powder.

ATOMET Powder	Lateral Surface		Top Surface	
	Ra (µm)	Wa (µm)	Ra (µm)	Wa (µm)
28	10.0 ± 0.9	3.7 ± 0.5	13.1 ± 1.4	2.3 ± 0.6
1001	13.7 ± 1.1	5.8 ± 0.6	22.9 ± 1.1	21.3 ± 1.5
4701	12.1 ± 1.3	2.9 ± 0.4	25.3 ± 1.4	13.7 ± 1.1
4801	5.4 ± 0.8	1.0 ± 0.1	10.5 ± 1.8	10.0 ± 1.5
Fe 99.8	11.4 ± 1.2	4.0 ± 0.4	20.1 ± 1.2	4.9 ± 0.7

**Table 5:** Cross-sectional porosity in the LENS-fabricated solid samples from the ATOMET and reference powders.

ATOMET and Reference Powder	Porosity LENS-Fabricated Sample (%)
28	4.2
1001	0.1
4701	0.03
4801	0.1
Fe 99.8	1.1
